# CLICK-ID: A novel dataset for Indonesian clickbait headlines

**DOI:** 10.1016/j.dib.2020.106231

**Published:** 2020-08-27

**Authors:** Andika William, Yunita Sari

**Affiliations:** Universitas Gadjah Mada, Indonesia

**Keywords:** Indonesian, Natural Language Processing, News articles, Clickbait, Text-classification

## Abstract

News analysis is a popular task in Natural Language Processing (NLP). In particular, the problem of clickbait in news analysis has gained attention in recent years [1, 2]. However, the majority of the tasks has been focused on English news, in which there is already a rich representative resource. For other languages, such as Indonesian, there is still a lack of resource for clickbait tasks. Therefore, we introduce the CLICK-ID dataset of Indonesian news headlines extracted from 12 Indonesian online news publishers. It is comprised of 15,000 annotated headlines with clickbait and non-clickbait labels. Using the CLICK-ID dataset, we then developed an Indonesian clickbait classification model achieving favourable performance. We believe that this corpus will be useful for replicable experiments in clickbait detection or other experiments in NLP areas.

## Specifications Table

**Table:** 

Subject	Computer Science
Specific subject area	Indonesian Language, Natural Language Processing, Text Classification
Type of data	Text files
How data were acquired	By scraping online news websites
Data format	Raw Analysed Filtered
Parameters for data collection	Articles were collected from the website's index pages of online news publishers. The data collected were the headline of the article, source publisher, published date and time, category, sub-category, and content of the article. Published date and time were separated into different columns. When a category or sub-category data is unavailable, it is left as blank.
Description of data collection	Articles were collected from a selected set of 12 Indonesian online news publishers with varying numbers. It was collected from each publisher using individual web scrapers. A sample of the articles, where the headlines are to be annotated, were then chosen accordingly from each publisher. In total, 15,000 sample headlines were annotated by undergraduate students into either clickbait or non-clickbait label.
Data source location	Articles were collected at Universitas Gadjah Mada, Indonesia.
Data accessibility	Repository name: Mendeley Data Data identification number: doi:10.17632/k42j7 × 2kpn.2 Direct URL to data: https://data.mendeley.com/datasets/k42j7X2kpn/1

## Value of the Data

• To our best knowledge, the CLICK-ID dataset is the largest clickbait dataset in the Indonesian language. It can provide a basis for further development on clickbait tasks in the Indonesian language.
• The CLICK-ID dataset is available in 3 versions that are based on the level of agreement between annotators which may help researchers to use more reliable annotations.
• In contrast with other clickbait datasets, the CLICK-ID dataset presents analysis on groups headlines based on their categories to give a perspective on how clickbait rates vary in different categories.
• The CLICK-ID dataset compiles headlines from 12 distinct online news media publishers and offers information to the common newsreaders of Indonesian media about which publishers have a higher tendency of publishing clickbait articles.
• The CLICK-ID dataset provides additional properties of the article. It can be extended for usage on various NLP tasks other than clickbait detection, such as text-categorization and training word embeddings.

## 1 Data Description

The CLICK-ID is a collection of Indonesian news headlines collected from 12 local Indonesian news publishers. The 12 publishers chosen are; detikNews, Fimela, Kapanlagi, Kompas, Liputan6, Republika, Sindonews, Tempo, Tribunnews, Okezone, Wowkeren, and Posmetro-Medan. The CLICK-ID corpus consists of 46,517 collected headlines that are divided into 2 groups, (i) 15,000 annotated headlines, and (ii) 31,517 non-annotated headlines. The annotated headlines are annotated with either clickbait or non-clickbait label, with the headline sentence serving as the only basis for judgment. Each headline is annotated by 3 annotators in which the majority is taken as ground truth. To offer a more thorough result of our annotation, we divided our annotated headlines into 3 files; *main, all_agree,* and *does_not_agree*. The *main* file contains the entire annotated dataset, whereas *all_agree* only contains headlines with annotations that were agreed by all annotators and *does_not_agree* only contains annotations with disagreements.

The files of CLICK-ID are available in 2 file extensions, comma-separated-values (.csv) and spreadsheet (.xlsx) file. It is stored in different folders with their contents corresponding to their folder names. In the repository, the CLICK-ID folder contains 2 folders; the raw folder and the annotated folder. Raw folders contain the original files that were generated as a result of the scraping process. On the other hand, the annotated folder contains the selected headlines that have been annotated.

All folders contain 2 folders of ‘csv’ and ‘xlsx’ which corresponds to the extension of the file inside. Files between these folders have identical contents. The files in the annotated folder differ in which they have an additional column for labels (‘label’ and ‘label_score’). The ‘label_score’ column in these files represents the same values as the ‘label’ column, where the clickbait label is represented as ‘1’ and non-clickbait as ‘0’. Furthermore, the spreadsheet files in the annotated folder contain 2 sheets. One with the labels and the other with the full details of the article (date, category, contents etc). There is also an additional folder named ‘combined’ in the annotated folder. It contains one *main* spreadsheet file along with the additional files of *main, all_agree,* and *does_not_agree* with (.csv) and (.json) extensions.

The details of the CLICK-ID dataset are highlighted in Table 1, where it was analysed using Microsoft Excel. Table 1 shows all the twelve publishers that were collected. In total, there are 46,119 articles collected (Article column), out of which 15,000 are sampled for annotation (Annotated columns). The 3 columns in the Annotated presents the results of our annotation. In total, our annotation shows there are 8710 non-clickbait and 6290 clickbait labels. Out of this total, annotations that were fully agreed upon (*all_agree* file) are comprised of 5297 non-clickbait and 3316 clickbait labels. In terms of reliability, our main dataset obtained a “moderate” inter-annotator agreement Fleiss’ K [3, 4] score of 0.42.

**Table 1.** CLICK-ID dataset. This table presents the distribution of headlines and their clickbait rates across publishers.

Publisher	Articles	Non-annotated	Annotated *(total, non-clickbait, clickbait)*		
detikNews	5468	4468	1000	890	110
fimela	788	88	700	306	394
kapanlagi	1006	6	1000	603	397
kompas	3243	1743	1500	1157	343
liputan6	4581	3081	1500	613	887
okezone	4664	3164	1500	741	759
posmetro	307	7	300	71	229
republika	5782	4282	1500	1267	233
sindonews	3572	2072	1500	1215	285
tempo	4026	2526	1500	1118	382
tribunnews	9662	8162	1500	451	1049
wowkeren	3020	1520	1500	278	1222

Total	46119	31119	15000	8710	6290

*posmetro-medan is referred as posmetro

**Fig 1:**
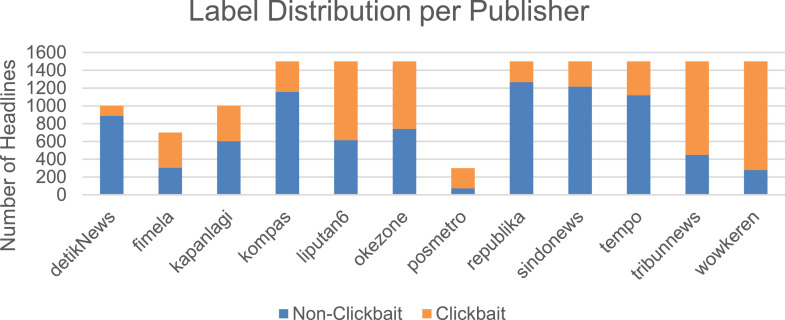
Distribution of headlines and their clickbait/non-clickbait labels per Publisher.

Clickbait headlines are detected in all publishers, with varying percentages. The distribution of the non-clickbait and clickbait headlines are visualized in Fig. 1. Here we see the different distributions of clickbait as presented in Table 1, in which it shows that detikNews have the lowest clickbait rate whereas wowkeren have the highest clickbait rate.

**Fig 2:**
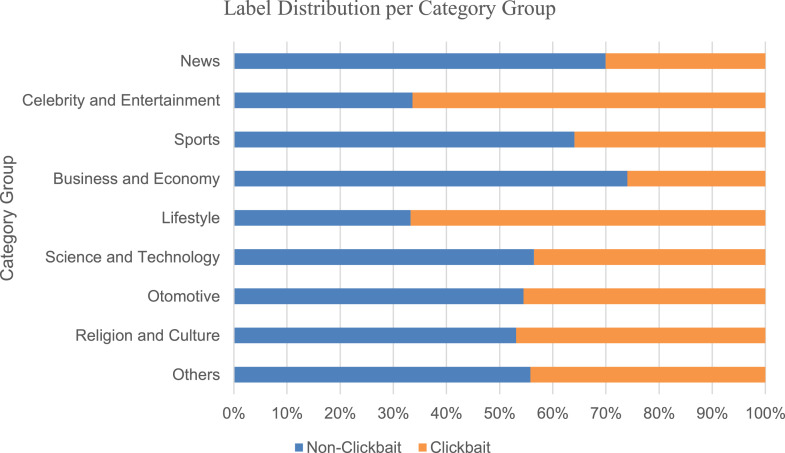
Distribution of label percentage per Category Group.

To analyze clickbait rates based on headlines’ categories, it is necessary to group the headlines first. However, since publishers use different category names, an attempt to group similar categories from each publisher was done. Namely, the categories from each publisher are grouped into one of the defined 9 Category Groups; *News, Celebrity & Entertainment, Sports, Business & Economy, Lifestyle, Science & Technology, Otomotive, Religion & Culture,* and *Others*. The details of which are presented in Table 2. Remaining categories that do not fit into any of these groups are classified into an additional Category Group *‘Others’*. The results of this grouping are shown in Table 3 shows the number of clickbait headlines of the combined articles belonging to the category group across publishers. Fig. 2 illustrates the distribution of clickbait headlines and comparison across Category Groups. This shows that the *Business & Economy* and *News* category groups have the lowest clickbait rates, while *Celebrity & Entertainment* and *Lifestyle* have the highest clickbait rates.

**Table 2.** Publishers that have categories grouped into the Category Groups; News, Celebrity & Entertainment, Sports, Business & Economy, Lifestyle, Science & Technology, Otomotive, Religion & Culture, Others. To analyze clickbait headlines based on their categories, they first need to be grouped. Since publishers have different category names for similar topics, the CLICK-ID dataset groups headlines into 9 general categories.

Publisher	Category								
	News	Celebrity & Entertainment	Sports	Business & Economy	Lifestyle	Science & Technology	Otomotive	Religion & Culture	Others
detikNews	✓	✓	-	-	-	-	-	-	-
Fimela	-	-	-	-	✓		-	-	-
kapanlagi	-	✓	-	-	-	-	-	-	-
kompas	✓	✓	✓	✓	✓	✓	✓	-	-
liputan6	✓	✓	✓	✓	✓	✓	✓	-	-
okezone	✓	✓	✓	✓	✓	✓	✓	✓	-
posmetro	✓	-	-	-	-	-	-	-	-
republika	✓	-	✓	✓	✓	✓	✓	✓	✓
sindonews	✓	-	✓	✓	✓	✓	✓	-	-
tempo	✓	✓	✓	✓	✓	✓	✓	-	✓
tribunnews	✓	✓	✓	✓	✓	✓	✓	✓	✓
wowkeren	✓	✓	-	-	✓	✓	-	-	✓

**Table 3.** Headline distribution per Category Group. Based on the categories that were grouped into the Category Groups, the distribution of clickbait headlines are calculated to be measured.

Category Groups	Category Headlines	Clickbait	Clickbait percentage
News	6379	1918	30.1%
Celebrity and Entertainment	3009	1998	66.4%
Sports	1727	620	35.9%
Business and Economy	1463	380	26.0%
Lifestyle	1315	878	66.8%
Science and Technology	411	179	43.6%
Otomotive	321	146	45.5%
Religion and Culture	194	91	46.9%
Others	181	80	44.2%
Total	15000	6290	41.9%

## 2 Experimental design, materials, and methods

In this section, the procedure of collecting, as well as pre-processing, of the CLICK-ID dataset is discussed. Furthermore, we discuss the experiment of using the dataset to develop an Indonesian clickbait classification model.

By the nature of the dataset, primary data is collected directly from published articles. Specifically, we focused on articles that were published in the Indonesian language. Several datasets have been compiled for the studies of clickbait in the English language [1, [5], [6], [7]]. The procedure of data collection for the CLICK-ID dataset is modelled based on these previous works, in particular Chakraborty [6]. We also improved and used the sources that have been implemented from previous works on compiling an Indonesian clickbait dataset by Maulidi and Ayilillahi [8, 9].

Since there is a large number of Indonesian publishers and it is not practical to collect from them all, only a selected few are chosen. The criteria are mostly based on popularity since the aim of the dataset is to best represent Indonesian articles. Nine of the publishers chosen are on the top 50 most visited websites in Indonesia based on Alexa's [10] ranking in September 2019, as shown in Table 4. From every publisher, all news categories were scraped from the publishers’ website. Only in the case of detik.com where only the ‘detikNews’ category group was scrapped.

**Table 4.** Top 50 most accessed website in Indonesia by Alexa [8].

Publisher	Source Website	Alexa Rank
detikNews	www.detik.com	5th
fimela	www.fimela.com	
kapanlagi	www.kapanlagi.com	19th
kompas	www.kompas.com	7th
liputan6	www.liputan6.com	6th
okezone	www.okezone.com	1st
posmetro	www.posmetro-medan.com	
republika	www.republika.co.id	
sindonews	www.sindonews.com	9th
tempo	www.tempo.co	25th
tribunnews	www.tribunnews.com	3rd
wowkeren	www.wowkeren.com	38th

Other considerations include the availability of data (some publishers only keep a limited number of past articles). Furthermore, to create a balanced dataset, several assumptions were made by the authors on how likely a publisher is to publish a clickbait based on their recent articles. Publishers that are assumed to be more likely to have clickbait articles are then also included. These considerations then made up into the final selection of the 12 publishers.

## 3 Scraping

Collection of the articles from the 12 publishers were done by scraping their respective websites. Specifically, the publishers’ index pages, where published articles from the past are kept, were scraped from. For efficiency, a specific range of date was targeted for all publishers. The articles that are scraped are the ones that were published during the dates of 10^th^-21^st^ September 2019. For each publisher, there is an individual scraper assigned. In cases where a publisher does not have an index page, as in the case of Posmetro-Medan, random available articles on the website are retrieved without regards as to the date it was published. Since publishers publish articles at different rates, the limited range of date results in different amounts of headlines scraped from each publisher.

The open-source Python web scraper library Scrapy [11] was utilized to extract data from the websites. Individual spiders are designed producing a (.csv) file for each publisher. Moreover, we conducted our scraping by following each website's ‘robots.txt’ scraping guideline to scrap within the allowed protocol of each website.

In general, the procedure of the spider follows the algorithm as laid out in Algorithm 1 with slightly different modifications to accommodate the different index page structures. First, it begins by navigating to the publisher's index page and parsing all the article URLs in the given page. The articles’ data are then collected from these article URLs. After all the articles data on an index page is collected, it then moves to the next page and the procedure is repeated. For each article, the spider collects 6 information regarding the article: headline, publisher name (source), date and time the article was published, category and sub-category of the article, the content of the article, and the URL of the article page. During this process, preliminary pre-processing was done to the data by removing irrelevant symbols such as html tags. In cases where the data is not provided by the publisher, it is left with the symbol ‘-‘. This procedure applies for most of the scraping process, except in cases where the publisher does not provide an index page. Finally, the scraping results in 12 distinct files each belonging to a publisher. In total, we collected 46,119 articles data from the 12 publishers

Algorithm 1 Scraping Procedure

**Table:** 

Input:	*U* - Url of publisher's main index page
Parameter:	*N -* Number of days to scrape
Output:	*D -* Article Dataset containing: Title, Source, Date, Time, Category, Sub-Category, Content

1:	Generate list of per date index URL pages *L* by concatenating main URL *U* with number of dates range *N*
2:	**for***every index page link***in***L***do**
3:	**procedure** ParseIndexPage()**:**
4:	Generate a list of article links *AL* in page
5:	**for***every article link***in** A*L***do**
6:	Collect all necessary article data *A* from an article page
7:	**yield** article data *A* to *D*
8:	**if***next page exists***then**
9:	Navigate to next page
10:	**call** ParseIndexPage()
11:	**Generate** dataset file *D*

## 4 Pre-processing

During the scraping phase, pre-processing was done only in removing unwanted characters from the data that were collected. This includes metacharacters and tags used for HTML such as ‘\n’ and ’\t’ that should not be in the headline string. This is done through Python functions and regex operations. There was no additional pre-processing that was done to the headlines. Headline structure is preserved as it is collected (with symbols and Uppercases) for annotation.

The (.csv) files that were generated from the scrapers are then converted into .xlsx using a separate python module. Both files are also concatenated with other respective files from other publishers to generate a combined version file. We provide this pre-processing result in the *raw* folder.

## 5 Annotation

Following the results of the scraping and pre-processing of 46,119 articles, we selected a limited number of articles to be annotated. From most of the publishers, 1500 articles are chosen for their headlines to be annotated as shown in Table 1. Other publishers with fewer articles, such as Fimela, Posmetro-Medan, and Kapanlagi are taken appropriately according to their numbers. Since detikNews is comprised of only one category (news) only a limited number is selected to avoid unbalancing the dataset.

The annotation process is done manually through spreadsheet applications such as Microsoft Excel and Google Spreadsheet. Twelve individual spreadsheet files with a total of 15,000 headlines are distributed among 17 undergraduate students, who are all native Indonesian speakers. Each file is distributed to 3 different annotators, with some annotators receiving more than one file. The spreadsheet files used for this process are modified to have extra columns for the labels: ‘clickbait’ and ‘non-clickbait’ column. The sheets are then replicated to have 3 more copies with each sheet reserved for an annotator. Annotators were instructed to enter the value ‘1’ on the column label which matches their judgment, as shown in Table 5. A headline would have a value of ‘1’ on the column ‘clickbait’ if it is judged as a clickbait, and vice versa. Labels were determined based only on the headline of the article, which follows the standards of other clickbait datasets [1, 5, 7]**.**

**Table 5.** Sample of the annotation file given to the annotator.

Headline (Original)	*Non-Clickbait*	*Clickbait*
Truck Hilang Kendali di China, 10 Orang Tewas (Truck Loses Control in China, 10 People Died)	1	
Inilah 3 Lokasi di Kota Medan dengan harga Rumah yang Fantastis! (These are the 3 Locations in Medan City with Fantastical Home prices!)		1

Before the files are distributed, the participating annotators were gathered for a briefing. The purpose is to establish a single idea of what constitutes as a clickbait. This include discussions on the definition of a clickbait continued with a review of random samples of headlines. Each sample is annotated together with the author as the final determiner. If there is disagreement on the label, the reasons are discussed until a full agreement is reached. Annotation is then conducted on participants personal computer through spreadsheet applications. After is it done, the file is then reviewed by the author. If there are blanks or duplicate labels, the files are returned to the annotator for review. The results of the annotation are available in the *annotated* folder.

## 6 Experiment

Using the annotated headlines from the CLICK-ID dataset, we developed an Indonesian clickbait classification model. The model was developed based on the works of English clickbait classification model [5], [6], [7]**.** In particular, we approached the problem to replicate the results of Agrawal [6], which indicated the favourable results of using Bi-LSTM architecture and the CNN architecture. Only in this case, we implemented the model using the CLICK-ID dataset.

The Bi-LSTM *(Bidirectional LSTM)* model is an expansion of the LSTM architecture first proposed in 1997 [12]. It belongs to the family of Recurrent Neural Networks which are suited for analyzing sequential data [13]. The Bi-LSTM model is similar to the LSTM model, with the difference that it processes sequential data both forwards and backwards. On the other hand, the CNN architecture is originally an architecture suited for image classification that has been shown to also work well on text data [14].

First, we implemented further pre-processing by lowercasing all the headline inputs. We then use different pre-processing with the data resulting in 3 different data. One with symbols, one without symbols, and the other with stemmed words. We then processed our *main* and *all_agree* dataset into these 3 pre-processing methods, resulting in a total of 6 datasets. Using these 6 datasets, each is used as input for the 2 models, Bi-LSTM and CNN. For comparison, Chakraborty's [5] dataset is also used as input. We then performed a 5-fold Cross-validation on the dataset, shown in Table 6. Accuracy is used as the evaluation metric in which it is defined as the number of Correct Predictions divided by Total Predictions.

**Table 6.** 5-fold Cross-Validation performance.

Dataset	Avg Acc	
	CNN	Bi-LSTM
MainWithSymbol	0.7639	0.7697
MainNoSymbol	0.7579	0.7779
MainStemmedWords	0.7112	0.7270
AGWithSymbol	0.8576	0.8832
AGNoSymbol	0.8781	0.8678
AGStemmedWords	0.7958	0.8125

AG = *all-agree*

## Declaration of Competing Interest

The authors declare that they have no known competing financial interests or personal relationships which have, or could be perceived to have, influenced the work reported in this article.

## Appendix Supplementary materials

Image, application 1

## References

[1] Potthast M., Köpsel S., Stein B., Hagen M. (2016). Clickbait detection. ECIR: Advances in Information Retrieval.

[2] Zheng H.-T., Chen J.-Y., Yao X., Sangaiah A.K., Jiang Y., Zhao C.-Z. (2018). Clickbait convolutional neural network. Symmetry.

[3] Fleiss J.L. (1971). Measuring nominal scale agreement among many raters. J. Psychol. Bull..

[4] J.L. Fleiss, J. Cohen, The equivalence of weighted kappa and the intraclass correlation coefficient as measures of reliability, J. Educ. Psychol. Measur. 10.1177/001316447303300309.

[5] Chakraborty A., Paranjape B., Kakarla S., Ganguly N. (2016). Stop clickbait: detecting and preventing clickbaits in online news media. Proceedings of the IEEE/ACM International Conference on Advances in Social Networks Analysis and Mining (ASONAM).

[6] Agrawal A. (2016). Clickbait detection using deep learning. Proceedings of the 2nd International Conference on Next Generation Computing Technologies (NGCT).

[7] Anand A., Chakraborty T., Park N., Jose J.M. (2017). We used neural networks to detect clickbaits: You won't believe what happened next!. Advances in Information Retrieval.

[8] Maulidi R., Ayilillahi M.F. (2018). Penerapan neural network backprogpagation untuk klasifikasi artikel clickbait. Seminar Nasional Sains dan Teknologi (SENASTEK).

[9] Habibie I. (2018). Identifikasi Judul Berita Clickbait Berbahasa Indonesia dengan Algoritma Long Short Term Memory (LSTM) Recurrent Neural Network’. *Skripsi*. Fakultas Ilmu Komputer dan Teknologi Informasi, Universitas Sumatera Utara, Medan.

[10] Alexa Internet (2019).

[11] Kouzis-Loukas D. (2016). Learning Scrapy.

[12] Hochreiter S., Schmidhuber J. (1997). Long short-term memory. Neural Comput..

[13] Goodfellow I., Bengio Y., Courville A. (2016). Deep Learning.

[14] Y. Kim. Convolutional neural networks for sentence classification. *Proceedings of the Conference on Empirical Methods in Natural Language Processing*. 10.3115/v1/D14-1181. 10.18653/v1/d16-1076 PMC5300751 28191551

